# Lipoprotein(a) and the Risk for Recurrent Atherosclerotic Cardiovascular Events Among Adults With CKD: The Chronic Renal Insufficiency Cohort (CRIC) Study

**DOI:** 10.1016/j.xkme.2023.100648

**Published:** 2023-05-05

**Authors:** Bharat Poudel, Robert S. Rosenson, Shia T. Kent, Vera Bittner, Orlando M. Gutiérrez, Amanda H. Anderson, Mark Woodward, Elizabeth A. Jackson, Keri L. Monda, Archna Bajaj, Lei Huang, Mayank Kansal, Mahboob Rahman, Jiang He, Paul Muntner, Lisandro D. Colantonio, CRIC Study Investigators

**Affiliations:** a Department of Epidemiology, University of Alabama at Birmingham, Birmingham, Alabama; b Mount Sinai Heart, Icahn School of Medicine at Mount Sinai, New York, New York; c Center for Observational Research, Amgen Inc., Thousand Oaks, California; d Division of Cardiovascular Disease, Department of Medicine, University of Alabama at Birmingham, Birmingham, Alabama; e Division of Nephrology, Department of Medicine, University of Alabama at Birmingham, Birmingham, Alabama; f Department of Epidemiology, Tulane University, New Orleans, Louisiana; g The George Institute for Global Health, Imperial College London, United Kingdom; h The George Institute for Global Health, University of New South Wales, Sydney, Australia; i Perelman School of Medicine, University of Pennsylvania, Philadelphia, Pennsylvania; j Department of Medicine, Division of Cardiology, University of Illinois-Chicago, Chicago, Illinois; k Department of Medicine, Case Western Reserve University School of Medicine, University Hospitals of Cleveland, Ohio

**Keywords:** Atherosclerotic cardiovascular disease events, chronic kidney disease, Chronic Renal Insufficiency Cohort, lipoprotein(a)

## Abstract

**Rationale & Objective:**

Many adults with chronic kidney disease (CKD) and atherosclerotic cardiovascular disease (ASCVD) have high lipoprotein(a) levels. It is unclear whether high lipoprotein(a) levels confer an increased risk for recurrent ASCVD events in this population. We estimated the risk for recurrent ASCVD events associated with lipoprotein(a) in adults with CKD and prevalent ASCVD.

**Study Design:**

Observational cohort study.

**Setting & Participants:**

We included 1,439 adults with CKD and prevalent ASCVD not on dialysis enrolled in the Chronic Renal Insufficiency Cohort study between 2003 and 2008.

**Exposure:**

Baseline lipoprotein(a) mass concentration, measured using a latex-enhanced immunoturbidimetric assay.

**Outcomes:**

Recurrent ASCVD events (primary outcome), kidney failure, and death (exploratory outcomes) through 2019.

**Analytical Approach:**

We used Cox proportional-hazards regression models to estimate adjusted HR (aHRs) and 95% CIs.

**Results:**

Among participants included in the current analysis (mean age 61.6 years, median lipoprotein(a) 29.4 mg/dL [25th-75th percentiles 9.9-70.9 mg/dL]), 641 had a recurrent ASCVD event, 510 developed kidney failure, and 845 died over a median follow-up of 6.6 years. The aHR for ASCVD events associated with 1 standard deviation (SD) higher log-transformed lipoprotein(a) was 1.04 (95% CI, 0.95-1.15). In subgroup analyses, 1 SD higher log-lipoprotein(a) was associated with an increased risk for ASCVD events in participants without diabetes (aHR, 1.23; 95% CI, 1.02-1.48), but there was no evidence of an association among those with diabetes (aHR, 0.99; 95% CI, 0.88-1.10, *P* comparing aHRs = 0.031). The aHR associated with 1 SD higher log-lipoprotein(a) in the overall study population was 1.16 (95% CI, 1.04-1.28) for kidney failure and 1.02 (95% CI, 0.94-1.11) for death.

**Limitations:**

Lipoprotein(a) was not available in molar concentration.

**Conclusions:**

Lipoprotein(a) was not associated with the risk for recurrent ASCVD events in adults with CKD, although it was associated with a risk for kidney failure.

**Plain Language Summary**

In the current analysis of 1,439 adults with chronic kidney disease (CKD) and prevalent atherosclerotic cardiovascular disease (ASCVD) not on dialysis enrolled in the Chronic Renal Insufficiency Cohort study, lipoprotein(a) [Lp(a)] mass concentration was not associated with the risk for recurrent ASCVD events. In subgroup analyses, higher Lp(a) was associated with an increased risk for recurrent ASCVD events in participants without diabetes, but there was no evidence of an association among those with diabetes. Higher Lp(a) was associated with an increased risk for kidney failure, an exploratory outcome, in the overall study population. Lp(a) mass concentration may not be useful to identify adults with CKD who have an increased risk for recurrent ASCVD events, although it may be useful as a risk marker for future kidney failure.

Adults with chronic kidney disease (CKD) who have prevalent atherosclerotic cardiovascular disease (ASCVD) have a very high risk for recurrent ASCVD events.^1^ Lipoprotein(a) [Lp(a)] is a risk factor for the development of ASCVD,^2^ and higher levels of Lp(a) have been associated with an increased risk for recurrent ASCVD events in adults without CKD.^3^ Novel agents including antisense oligonucleotides and small interfering RNA targeting apolipoprotein(a) have been shown to reduce Lp(a) levels, and there are currently ongoing clinical trials to show their efficacy to prevent recurrent events in adults with ASCVD without severe kidney disease (NCT04023552 and NCT05581303).^4^^,^^5^ It is currently unclear whether high Lp(a) levels confer an increased risk for recurrent ASCVD events specifically in adults with CKD. If high Lp(a) levels are associated with an increased risk for recurrent ASCVD events in adults with CKD, this may represent an opportunity for ASCVD risk reduction in this high-risk population through novel Lp(a)-lowering therapies.^6^

The goal of the current study was to determine the association of Lp(a) with the risk for recurrent ASCVD events in adults with CKD. To accomplish this goal, we used data from the Chronic Renal Insufficiency Cohort (CRIC) study.

## Methods

### Study Population

The CRIC study enrolled 3,939 adults 21-74 years of age with mild-to-advanced CKD between May 2003 and August 2008 at 7 centers in the United States.^7^ Mild-to-advanced CKD was defined as an estimated glomerular filtration rate (eGFR) of 20-70 mL/min/1.73 m^2^ for adults aged 21-44 years, 20-60 mL/min/1.73 m^2^ for adults aged 45-64 years, and 20-50 mL/min/1.73 m^2^ for adults aged 65-74 years.^8^ Participants receiving dialysis were not included in the CRIC study. The CRIC study protocol was approved by the Institutional Review Boards at the participating centers, and all participants provided written informed consent.

All CRIC study participants completed an in-person study visit at baseline during which trained staff conducted an interview, physical examination, medication inventory, and collected blood and urine samples following standardized protocols. We included CRIC study participants who had a history of ASCVD at baseline, defined by a history of coronary heart disease (CHD), ischemic stroke or peripheral artery disease (PAD) (n = 1,477). We restricted the analysis to participants with Lp(a) data at baseline (n = 1,447). We excluded an additional 8 participants who did not have follow-up information after baseline, resulting in a final sample size of 1,439 participants (Figure S1).

### Lp(a) Measurements and Other Baseline Characteristics

Lp(a) mass concentration was measured during the baseline study visit using blood samples and a latex-enhanced immunoturbidimetric assay (Pointe Scientific).^9^ History of CHD was defined by self-report of a prior myocardial infarction (MI) or coronary revascularization. History of ischemic stroke was defined by self-report. History of PAD was defined by self-report of a prior diagnosis or by an ankle brachial index <0.9 during the baseline study visit.

Age, sex, income, education, current smoking, physical activity, antihypertensive medication use, and aspirin use were ascertained by self-report. We used data on race/ethnicity inferred through the ITMAT/Broad/CARe single nucleotide polymorphisms array.^10^ Body mass index was calculated as participant’s weight in kilograms divided by their height in meters squared. Baseline systolic blood pressure was measured 3 times and averaged. Diabetes was defined as fasting glucose ≥126 mg/dL, nonfasting glucose ≥200 mg/dL, or use of glucose-lowering medications. eGFR was calculated from serum creatinine, serum cystatin-C, age, and sex using the 2021 Chronic Kidney Disease Epidemiology Collaboration (CKD-EPI) equation (Table S1).^11^ Low-density lipoprotein (LDL) cholesterol was measured using β-quantification. Non-Lp(a) LDL cholesterol was calculated as LDL cholesterol – Lp(a) × 0.3.^12^ High-density lipoprotein cholesterol, triglycerides, high-sensitivity C-reactive protein, homocysteine, and fibroblast growth factor 23 were measured in blood samples. Urine albumin and creatinine were measured and used to calculate the albumin-creatinine ratio. Statin use was identified based on the baseline medication inventory.

### Outcomes

Participants were followed through annual study examinations and phone calls to identify possible study outcomes, including MI, ischemic stroke and PAD hospitalizations, kidney transplant, and dialysis. Medical records were retrieved and adjudicated by at least 2 study clinicians to confirm the occurrence of study outcomes. MI hospitalization was defined by symptoms, cardiac biomarker levels, and electrocardiograms consistent with acute myocardial ischemia. Ischemic stroke hospitalization was defined by sudden neurologic symptoms and neuroimaging consistent with acute cerebral infarction. PAD hospitalization was defined by a peripheral artery bypass or angioplasty or major amputation due to occlusive PAD. Deaths of CRIC study participants were identified from reports of relatives, retrieval of death certificates or obituaries, hospital and outpatient records, the Social Security Death Master File, and the National Death Index. Using death certificates, CHD death was defined by an *International Classification of Diseases, Tenth Revision* code of I20.xx-I25.xx and ischemic stroke death was defined by an *International Classification of Diseases, Tenth Revision* code of I63.xx.

The primary outcome of ASCVD was defined as the composite of MI hospitalization, ischemic stroke hospitalization, PAD hospitalization, CHD death, or ischemic stroke death. The following components of the primary outcome were investigated as secondary outcomes:

1) CHD event, defined as an MI hospitalization or CHD death.
2) MI hospitalization.
3) Ischemic stroke event, defined as an ischemic stroke hospitalization or ischemic stroke death.
4) Ischemic stroke hospitalization.
5) PAD hospitalization.

Also, we analyzed 2 exploratory outcomes, kidney failure, defined as kidney transplant or dialysis initiation, and death. Follow-up data through January 2019 were available for the current analysis.

### Statistical Analysis

We calculated summary statistics for characteristics of participants, overall and by quartiles of the Lp(a) distribution. Also, we calculated the median Lp(a) by participant characteristics.

We calculated the rate of ASCVD events and used an unadjusted Cox proportional-hazards regression model and restricted cubic splines to identify possible thresholds in the association of log-transformed Lp(a) with ASCVD events. The log-transformation was used because Lp(a) values are skewed to the right. We used the log-likelihood ratio test to determine whether restricted cubic splines departed from a log-linear association. We confirmed that the proportional-hazards assumption was not violated using log(-log(survival)) plots. We also calculated the unadjusted cumulative incidence of ASCVD events by quartiles of Lp(a) after accounting for the competing risk for death.^13^

We used 3 Cox proportional-hazards regression models with progressive adjustment for covariates to estimate hazard ratios (HRs) and 95% confidence intervals (CIs) for recurrent ASCVD events associated with 1 standard deviation (SD) higher log-Lp(a) and by quartiles of Lp(a). Model 1 adjusted for age, sex, race/ethnicity, CRIC study center, education, and income. Model 2 included adjustment for the variables in Model 1 and smoking status, physical activity, body mass index, diabetes, systolic blood pressure, antihypertensive medication use, eGFR, high-sensitivity C-reactive protein, and albumin-creatinine ratio. Model 3 included adjustment for the variables in Model 2 and high-density lipoprotein cholesterol, triglycerides, fibroblast growth factor 23, homocysteine, use of aspirin and statins, and non-Lp(a) LDL cholesterol.

The risk for ASCVD events associated with Lp(a) may differ by participant characteristics.^14^ Therefore, we conducted prespecified analyses to calculate HRs and 95% CIs for recurrent ASCVD events associated with 1 SD higher log-Lp(a) including adjustment for variables in Model 3 within subgroups defined by age, sex, race/ethnicity, and variables contributing to an individual’s ASCVD risk: diabetes, systolic blood pressure, history of CHD, stroke, and PAD, eGFR, albumin-creatinine ratio, LDL cholesterol, high-sensitivity C-reactive protein, and use of aspirin and statins. In addition to the main effects, we included interaction terms between the stratification variable (eg, male vs female) and all variables in the model to determine whether the association between Lp(a) and recurrent ASCVD events differed across the subgroups.^15^

We used Cox proportional-hazards regression with adjustment in the 3 models described above to estimate HRs and 95% CIs associated with Lp(a) for the secondary outcomes of CHD event, MI hospitalization, ischemic stroke event, ischemic stroke hospitalization, and PAD hospitalization, and for the exploratory outcomes of kidney failure and death. We repeated the subgroup analyses described above for recurrent ASCVD events for kidney failure and death. We did not conduct subgroup analyses for the secondary outcomes as the number of events were expected to be low in some subgroups.

To include participants with missing data (itemized in Table S2) in regression models, we used multiple imputation by chained equations. Specifically, we created 20 datasets replacing missing data with estimates predicted based on observed values from all the covariates included in Model 3 and the outcomes.^16^^,^^17^ Results across imputed datasets were combined using Rubin’s rules.^18^ Statistical analyses were performed using SAS version 9.4 software (SAS Institute) and a 2-sided level of significance of 0.05.

## Results

The mean age for participants included in the current analysis was 61.6 years; 58.5% were men and 62.3% had diabetes. The median Lp(a) mass concentration was 29.4 mg/dL (25^th^, 75^th^ percentiles 9.9, 70.9 mg/dL). Table S3 shows the median Lp(a) by levels of participant characteristics. Participants with higher Lp(a) levels were more likely to be of Black race/ethnicity, less likely to be male, and had lower non-Lp(a) LDL cholesterol levels (Table 1).

**Table 1:** Characteristics of Chronic Renal Insufficiency Cohort Study Participants Included in the Current Analysis, Overall and by Quartiles of Lp(a) Mass Concentration.

Participant characteristics	Overall	Quartiles of Lp(a)			
		Quartile 1	Quartile 2	Quartile 3	Quartile 4
N	1,439	360	360	360	359
Range of Lp(a), mg/dL	1.5 to 261.5	1.5 to <9.9	9.9 to <29.4	29.4 to <70.9	70.9 to 261.5
Age (y), mean ± SD	61.6 ± 8.5	61.4 ± 9.1	61.5 ± 8.8	62.0 ± 7.7	61.7 ± 8.1
Sex, male, n (%)	842 (58.5%)	236 (65.6%)	213 (59.2%)	207 (57.5%)	186 (51.8%)
Race/ethnicity, n (%)					
White	533 (40.8%)	213 (63.6%)	154 (46.4%)	84 (26.6%)	82 (25.4%)
Black	604 (46.2%)	56 (16.7%)	128 (38.6%)	207 (65.5%)	213 (65.9%)
Other	169 (12.9%)	66 (19.7%)	50 (15.1%)	25 (7.9%)	28 (8.7%)
Study center, n (%)					
University of Pennsylvania	188 (13.1%)	37 (10.3%)	52 (14.4%)	49 (13.6%)	50 (13.9%)
Johns Hopkins University	212 (14.7%)	41 (11.4%)	41 (11.4%)	63 (17.5%)	67 (18.7%)
Case Western Reserve University	196 (13.6%)	60 (16.7%)	47 (13.1%)	42 (11.7%)	47 (13.1%)
University of Michigan	175 (12.2%)	49 (13.6%)	39 (10.8%)	40 (11.1%)	47 (13.1%)
University of Illinois at Chicago	337 (23.4%)	80 (22.2%)	88 (24.4%)	97 (26.9%)	72 (20.1%)
Tulane University Health Science Center	180 (12.5%)	48 (13.3%)	47 (13.1%)	41 (11.4%)	44 (12.3%)
Kaiser Permanente of Northern California	151 (10.5%)	45 (12.5%)	46 (12.8%)	28 (7.8%)	32 (8.9%)
Income, n (%)					
≤$20,000	550 (45.3%)	115 (37.3%)	150 (48.1%)	140 (47.8%)	145 (48.3%)
$20,001-$50,000	354 (29.2%)	80 (26.0%)	95 (30.4%)	88 (30.0%)	91 (30.3%)
$50,001-$100,000	216 (17.8%)	71 (23.1%)	52 (16.7%)	45 (15.4%)	48 (16.0%)
>$100,000	93 (7.7%)	42 (13.6%)	15 (4.8%)	20 (6.8%)	16 (5.3%)
Education less than high school, n (%)	364 (25.3%)	68 (18.9%)	84 (23.3%)	105 (29.2%)	107 (29.8%)
Current smoking, n (%)	224 (15.6%)	45 (12.5%)	40 (11.1%)	71 (19.7%)	68 (18.9%)
Physical activity, total MET score, median (25th, 75th percentile)	142.3 (93.1, 215.8)	146.4 (106.8, 229.9)	141.7 (81.5, 214.8)	142.2 (89.5, 216.1)	139.7 (87.8, 202.3)
Body mass index (kg/m^2^), n (%)					
<25	195 (13.6%)	45 (12.6%)	46 (12.9%)	42 (11.7%)	62 (17.3%)
25 to <30	399 (27.8%)	113 (31.7%)	89 (24.9%)	104 (28.9%)	93 (25.9%)
≥30	839 (58.5%)	199 (55.7%)	222 (62.2%)	214 (59.4%)	204 (56.8%)
Diabetes, n (%)	897 (62.3%)	227 (63.1%)	231 (64.2%)	223 (61.9%)	216 (60.2%)
Systolic blood pressure, mm Hg, mean ± SD	131.6 ± 23.7	127.3 ± 22.1	129.3 ± 21.9	133.6 ± 23.5	136.3 ± 26.0
Antihypertensive medication use, n (%)	1316 (92.0%)	322 (89.9%)	331 (92.2%)	326 (91.3%)	337 (94.4%)
History of CHD, n (%)	838 (58.2%)	222 (61.7%)	213 (59.2%)	194 (53.9%)	209 (58.2%)
History of stroke, n (%)	381 (26.5%)	81 (22.5%)	91 (25.3%)	110 (30.6%)	99 (27.6%)
History of PAD, n (%)	749 (52.1%)	169 (46.9%)	180 (50.0%)	191 (53.1%)	209 (58.2%)
eGFR, mL/min/1.73 m^2^, mean ± SD	42.1 ± 16.0	45.3 ± 16.5	42.8 ± 16.3	40.7 ± 16.2	39.5 ± 14.6
hs-CRP ≥2 mg/L, n (%)	887 (61.6%)	206 (57.2%)	215 (59.7%)	246 (68.3%)	220 (61.3%)
ACR, n (%)					
<30 mg/g	517 (37.1%)	135 (38.8%)	135 (38.9%)	128 (36.1%)	119 (34.6%)
30-300 mg/g	412 (29.6%)	114 (32.8%)	101 (29.1%)	99 (27.9%)	98 (28.5%)
>300-1,000 mg/g	195 (14.0%)	45 (12.9%)	45 (13.0%)	58 (16.3%)	47 (13.7%)
>1,000 mg/g	270 (19.4%)	54 (15.5%)	66 (19.0%)	70 (19.7%)	80 (23.3%)
Fibroblast growth factor 23, RU/mL, median (25th, 75th percentile)	172.0 (112.8, 284.4)	161.7 (107.1, 265.8)	179.8 (115.3, 309.6)	177.1 (114.4, 297.0)	182.2 (114.4, 267.3)
HDL cholesterol, mg/dL, mean ± SD	45.5 ± 13.7	42.6 ± 12.0	43.9 ± 12.8	46.0 ± 14.3	49.5 ± 14.7
Triglycerides, mg/dL, median (25th, 75th percentile)	132.0 (95.0, 191.0)	150.0 (108.0, 220.0)	137.0 (102.0, 207.5)	122.0 (86.5, 166.0)	123.0 (90.0, 170.0)
Homocysteine, μmol/L, median (25th, 75th percentile)	15.2 (12.3, 18.9)	14.7 (12.2, 17.9)	15.2 (12.5, 19.4)	15.3 (11.9, 19.2)	15.7 (12.5, 19.6)
Aspirin use	844 (59.1%)	218 (60.7%)	221 (61.6%)	204 (57.0%)	201 (57.1%)
Statin use	1,033 (72.3%)	267 (74.4%)	254 (70.8%)	252 (70.4%)	260 (73.9%)
LDL cholesterol, mg/dL, mean ± SD	96.9 ± 35.0	88.0 ± 31.3	91.2 ± 32.1	99.1 ± 34.5	109.5 ± 37.9
Non-Lp(a) LDL cholesterol, mg/dL, mean ± SD	83.4 ± 34.0	86.5 ± 31.3	85.7 ± 32.0	84.7 ± 34.3	76.5 ± 37.5

Abbreviations: ACR, albumin-to-creatinine ratio; CHD, coronary heart disease; eGFR, estimated glomerular filtration rate; HDL, high-density lipoprotein; hs-CRP, high-sensitivity C-reactive protein; LDL, low-density lipoprotein; Lp(a), lipoprotein(a); MET, metabolic equivalent; PAD, peripheral artery disease; SD, standard deviation.

**Figure 1:**
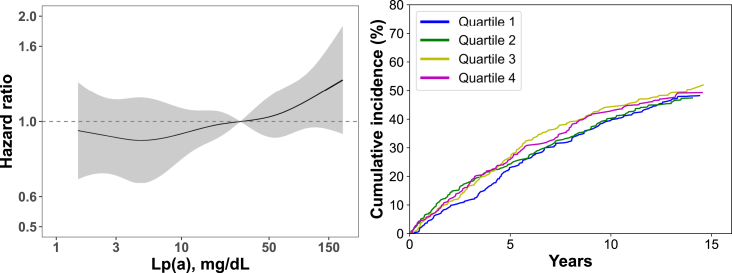
Association between Lp(a) and recurrent atherosclerotic cardiovascular events. Abbreviation: Lp(a), lipoprotein(a). The panel on the left shows unadjusted hazard ratios (black line) and 95% confidence intervals (gray area) for recurrent atherosclerotic cardiovascular events associated with Lp(a) levels using restricted cubic splines. The *P* value assessing whether hazard ratios associated with Lp(a) depart from a linear association (ie, a straight line) was 0.73. The panel on the right shows the unadjusted cumulative incidence of recurrent atherosclerotic cardiovascular events associated with quartiles of Lp(a) accounting for the competing risk of death. Range of Lp(a) values in each quartile: Quartile 1: 1.5 to <9.9 mg/dL; Quartile 2: 9.9 to <29.4 mg/dL; Quartile 3: 29.4 to <70.9 mg/dL; Quartile 4: 70.9 to 261.5 mg/dL.

Over a median follow-up of 6.6 years, 641 participants experienced a recurrent ASCVD event. There was no evidence suggesting a possible threshold above which Lp(a) levels were associated with a higher risk for ASCVD events using restricted cubic splines (Fig 1, left panel), or by comparing the cumulative incidence of ASCVD events by quartiles of Lp(a) (Fig 1, right panel). After full adjustment, the HR for ASCVD events for each 1 SD higher level of log-Lp(a) was 1.04 (95% CI, 0.95-1.15; Table 2). The fully adjusted HRs for ASCVD events associated with the second, third, and fourth quartile versus the first quartile of the Lp(a) distribution were 1.00 (95% CI, 0.79-1.27), 1.06 (95% CI, 0.83-1.36) and 1.06 (95% CI, 0.82-1.37), respectively (Table S4).

**Table 2:** Incidence Rate and Hazard Ratio for Recurrent Atherosclerotic Cardiovascular Events Associated With Lp(a) Levels.

	Recurrent ASCVD events
Events/person-years	641/10,585
Rate (95% CI) per 1,000 person-years	60.5 (55.9-65.2)
Hazard ratio (95% CI) per 1 SD higher log-transformed Lp(a)	
Model 1	1.07 (0.98-1.17)
Model 2	1.03 (0.94-1.12)
Model 3	1.04 (0.95-1.15)

*Note*: Recurrent ASCVD events include myocardial infarction hospitalization, ischemic stroke hospitalization, peripheral artery disease hospitalization, coronary heart disease death or ischemic stroke death.

The SD of log-transformed Lp(a) was equal to 1.3 units, which represents a 3.7 times increase in Lp(a) levels in their original scale.

Model 1 includes adjustment for age, sex, Chronic Renal Insufficiency Cohort study center, race/ethnicity, education and income.

Model 2 include adjustment for variables in Model 1 and smoking status, physical activity, body mass index, diabetes, systolic blood pressure, antihypertensive medication use, estimated glomerular filtration rate, high-sensitivity C-reactive protein, and albumin-creatinine ratio.

Model 3 includes adjustment for variables in Model 2 and high-density lipoprotein cholesterol, triglycerides, fibroblast growth factor 23, homocysteine, use of aspirin and statins, and non-Lp(a) low-density lipoprotein cholesterol.

Abbreviations: ASCVD, atherosclerotic cardiovascular disease; CI, confidence interval; Lp(a), lipoprotein(a); SD, standard deviation.

**Figure 2:**
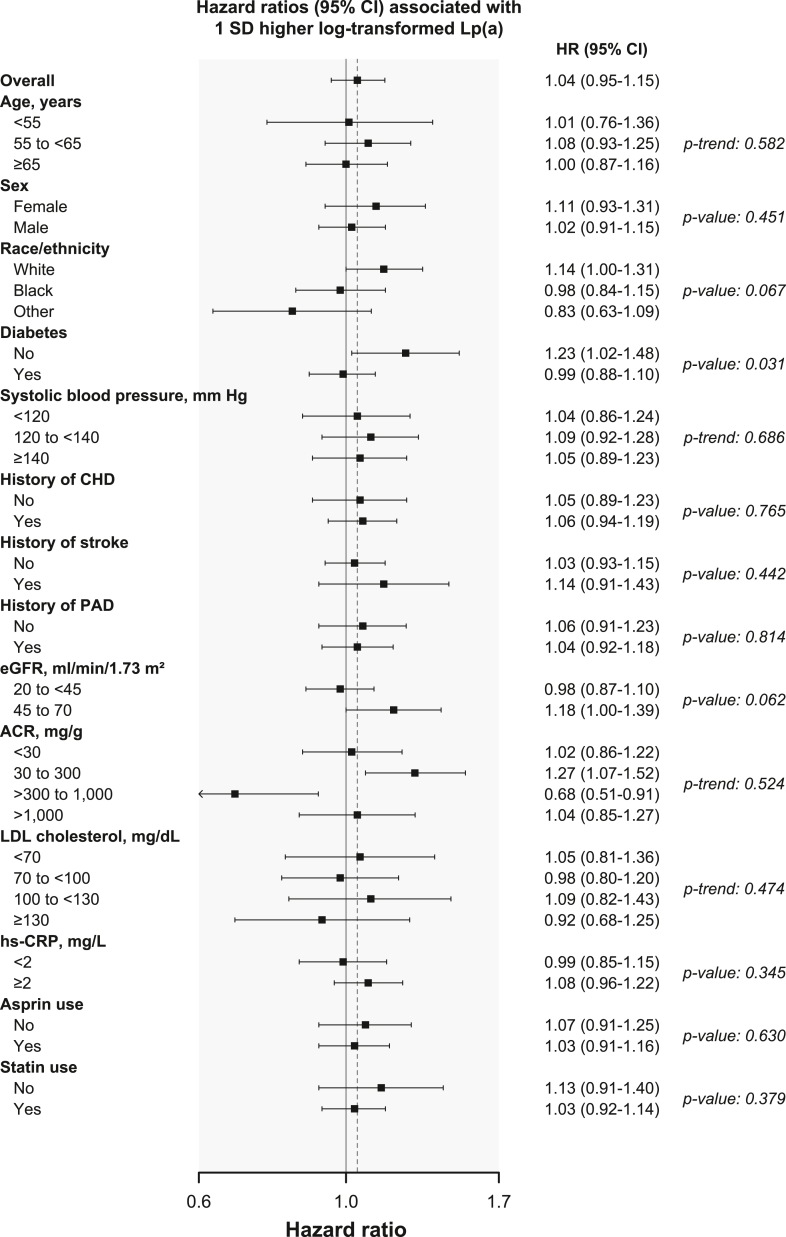
Hazard ratios for recurrent atherosclerotic cardiovascular events associated with 1 standard deviation higher log-transformed Lp(a) within subgroups. Abbreviations: ACR, albumin-creatinine-ratio; CHD, coronary heart disease; CI, confidence interval; eGFR, estimated glomerular filtration rate; HR, hazard ratio; hs-CRP, high-sensitivity C-reactive protein; LDL, low-density lipoprotein; Lp(a), lipoprotein(a); PAD, peripheral artery disease; SD, standard deviation. Recurrent atherosclerotic cardiovascular events include myocardial infarction hospitalization, ischemic stroke hospitalization, PAD hospitalization, CHD death, or ischemic stroke death. The SD of log-transformed Lp(a) was equal to 1.3 units, which represents a 3.7 times increase in Lp(a) levels in their original scale. All hazard ratios were adjusted for age, sex, race/ethnicity, Chronic Renal Insufficiency Cohort study center, education, income, smoking status, physical activity, body mass index, diabetes, systolic blood pressure, antihypertensive medication use, eGFR, high-density lipoprotein cholesterol, triglycerides, high-sensitivity C-reactive protein, albumin-creatinine ratio, fibroblast growth factor 23, homocysteine, use of aspirin and statins, and non-Lp(a) LDL cholesterol.

In subgroup analyses, the fully adjusted HR for recurrent ASCVD events per each 1 SD higher log-Lp(a) was 0.99 (95% CI, 0.88-1.10) and 1.23 (95% CI, 1.02-1.48) among participants with and without diabetes, respectively (*P* comparing HRs = 0.034; Fig 2). There was no evidence of a difference in the association between Lp(a) and recurrent ASCVD events across the strata for the other subgroups investigated.

During follow-up, 491 participants experienced a CHD event (ie, MI hospitalization or CHD death), 286 had an MI hospitalization, 126 had an ischemic stroke event (ie, ischemic stroke hospitalization or ischemic stroke death), 111 had an ischemic stroke hospitalization, 153 had a PAD hospitalization, 510 developed kidney failure, and 845 died. There was no evidence of an association between log-Lp(a) and the risk for CHD event, MI hospitalization, ischemic stroke event, ischemic stroke hospitalization, PAD hospitalization, or death (Table 3). The fully adjusted HR for kidney failure associated with 1 SD higher log-Lp(a) was 1.16 (95% CI, 1.04-1.28). There was no evidence of an association between quartiles of Lp(a) and CHD event, MI hospitalization, ischemic stroke event, ischemic stroke, PAD hospitalization, and death (Table S5). After full multivariable adjustment, the HRs for kidney failure associated with the second, third, and fourth quartiles versus the first quartile of the Lp(a) distribution were 1.24 (95% CI, 0.94-1.65), 1.61 (95% CI, 1.22-2.14), and 1.33 (95% CI, 1.00-1.77), respectively.

**Table 3:** Risk for Recurrent Cardiovascular Events (Secondary Outcomes), and Kidney Failure and Death (Exploratory Outcomes) Associated With Lp(a) Levels (Secondary Outcomes).

	Coronary Heart Disease Events	Myocardial Infarction Hospitalizations	Ischemic Stroke Events	Ischemic Stroke Hospitalizations	Peripheral Artery Disease Hospitalization	Kidney Failure	Death
Events/person-years	491/11,486	286/11,486	126/12,021	111/12,021	153/11,832	510/10,840	845/13,138
Rate (95% CI) per 1,000 person-years	42.7 (38.9-46.5)	24.9 (22.0-27.8)	10.5 (8.7-12.3)	9.2 (7.5-11.0)	12.9 (10.9-15.0)	47.0 (43.0-51.1)	64.3 (60.0-68.7)
Hazard ratio (95% CI) per 1 SD higher log-transformed Lp(a)							
Model 1	1.01 (0.91-1.12)	1.05 (0.92-1.19)	1.06 (0.85-1.31)	1.04 (0.84-1.30)	1.10 (0.92-1.31)	1.21 (1.09-1.34)	1.06 (0.99-1.15)
Model 2	0.97 (0.87-1.08)	1.01 (0.88-1.15)	1.05 (0.84-1.31)	1.05 (0.84-1.30)	1.05 (0.87-1.26)	1.12 (1.02-1.25)	1.01 (0.93-1.09)
Model 3	1.00 (0.90-1.11)	1.05 (0.92-1.21)	1.09 (0.86-1.37)	1.09 (0.87-1.37)	1.05 (0.87-1.27)	1.16 (1.04-1.28)	1.02 (0.94-1.11)

*Note*: The SD of log-transformed Lp(a) was equal to 1.3 units, which represents a 3.7 times increase in Lp(a) levels in their original scale.

Recurrent cardiovascular events include coronary heart disease events (ie, myocardial infarction hospitalization or coronary heart disease death), myocardial infarction hospitalizations, ischemic stroke events (ie, ischemic stroke hospitalization or ischemic stroke death), ischemic stroke hospitalizations, peripheral artery disease hospitalization.

Model 1 includes adjustment for age, sex, race/ethnicity, Chronic Renal Insufficiency Cohort study center, education and income.

Model 2 includes adjustment for variables in Model 1 and smoking status, physical activity, body mass index, diabetes, systolic blood pressure, antihypertensive medication use, estimated glomerular filtration rate, high-sensitivity C-reactive protein, and albumin-creatinine ratio.

Model 3 includes adjustment for variables in Model 2 and high-density lipoprotein cholesterol, triglycerides, fibroblast growth factor 23, homocysteine, use of aspirin and statins, and non-Lp(a) low-density lipoprotein cholesterol.

Abbreviations: CI, confidence interval; Lp(a), lipoprotein(a); SD, standard deviation.

There was no evidence of a difference in the risk for kidney failure associated with Lp(a) across subgroups defined by age, sex, race/ethnicity, diabetes, systolic blood pressure, history of CHD, stroke, or PAD, eGFR, albumin-creatinine ratio, high-sensitivity C-reactive protein, aspirin use, or statin use. The HR for kidney failure associated with 1 SD higher log-Lp(a) was 1.13 (95% CI, 0.86-1.50), 1.28 (95% CI, 0.99-1.67), 1.52 (95% CI, 1.07-2.16) and 0.81 (95% CI, 0.59-1.12) among participants with LDL cholesterol <70 mg/dL, 70 to <100 mg/dL, 100 to <130 mg/dL, and ≥130 mg/dL, respectively (*P* for the trend of HRs across LDL cholesterol categories = 0.043, Fig S2). In subgroup analyses, 1 SD higher log-Lp(a) was associated with an increased risk for death among participants with eGFR ≥45 mL/min/1.73 m^2^ (HR, 1.16; 95% CI, 1.00-1.35), but there was no evidence of an association among those with eGFR <45 mL/min/1.73 m^2^ (HR, 0.95; 95% CI, 0.86-1.04; *P* comparing HRs = 0.026, Fig S3). There was no evidence of an association between Lp(a) and the risk for death in any of the other subgroups analyzed.

## Discussion

In the current analysis of adults with CKD who had a history of ASCVD, there was no evidence of an association between Lp(a) and the risk for recurrent ASCVD events or death in the overall study population. There was also no evidence that Lp(a) levels were associated with the risk for CHD, ischemic stroke, or PAD events, separately. In an exploratory analysis, higher Lp(a) mass concentration was associated with an increased risk for kidney failure.

Prior studies combining participants with CKD with and without a history of ASCVD have shown that high Lp(a) levels are associated with the progression of atherosclerosis and an increased risk for ASCVD events.^9^^,^^19^^,^^20^ In a prior analysis of CRIC study participants with and without a history of ASCVD by Bajaj et al,^9^ the multivariable-adjusted HR for MI hospitalization associated with the highest quartile of Lp(a) versus the second lowest quartile (ie, the quartile with the lowest MI event rate) was 1.49 (95% CI, 1.05-2.11). However, these prior studies did not report the risk for ASCVD events associated with Lp(a) restricted to participants who had ASCVD. The lack of an association in the current study of adults with CKD and prevalent ASCVD suggests that higher Lp(a) mass concentration may not substantially increase the risk for recurrent events in this very high-risk population. Whether lowering Lp(a) through novel pharmacologic therapies reduces the risk for recurrent ASCVD events is currently under investigation. Results from the current study highlight the need for clinical trials to determine the benefit of novel Lp(a)-lowering therapies to reduce the risk for recurrent ASCVD events specifically in adults with CKD.

The high prevalence of diabetes among CRIC study participants included in the current analysis (62.3%) may have contributed to attenuating an association between Lp(a) and the risk for recurrent ASCVD events. In the current analysis, a higher Lp(a) mass concentration was associated with an increased risk for recurrent ASCVD events in participants without diabetes, but there was no association among those with diabetes. This is consistent with other prior studies suggesting that diabetes and metabolic syndrome may attenuate an association between Lp(a) and ASCVD risk.^21^, ^22^, ^23^, ^24^, ^25^, ^26^, ^27^ Although the exact mechanisms through which diabetes may attenuate an association between Lp(a) and ASCVD risk remain unclear, it has been speculated that this attenuation may be explained by differences in the characteristics of Lp(a) particles and their metabolism.^21^ Lp(a) consists of an LDL particle with its molecule of apolipoprotein B100 linked to a glycoprotein of highly variable size and weight known as apolipoprotein(a).^28^ Adults with diabetes have larger apolipoprotein(a) isoforms versus those without diabetes, a difference that may be caused by the effects of high insulin levels on the synthesis of apolipoprotein(a) in the liver.^22^^,^^29^^,^^30^ Lp(a) particles with large apolipoprotein(a) isoforms are catabolized faster than particles with smaller apolipoprotein(a) isoforms.^31^ Prior studies have shown that larger apolipoprotein(a) isoforms are associated with a lower risk for ASCVD events versus smaller isoforms.^32^, ^33^, ^34^, ^35^

Current scientific statements recommend reporting Lp(a) using standardized molar concentration as this may provide values that are comparable across different labs and study populations, improving the clinical interpretation of the risk associated with Lp(a).^36^^,^^37^ Lp(a) molar concentration may also be a better marker of the risk for ASCVD events than apolipoprotein(a) size or Lp(a) mass concentration.^28^^,^^38^ In a prior study conducted in Iceland, higher Lp(a) molar concentration was associated with a lower apolipoprotein(a) size and an increased risk for CHD events.^32^ After accounting for Lp(a) molar concentration, apolipoprotein(a) size was not associated with further CHD risk. Future studies should determine whether Lp(a) molar concentration is associated with the risk for recurrent ASCVD events in adults with CKD and prevalent ASCVD before concluding that Lp(a) is not useful for ASCVD risk stratification in this population.

In a prior analysis of the CRIC study combining participants with and without ASCVD followed for a median of 4.1 years, the multivariable-adjusted HR for kidney failure or 50% decline in eGFR associated with 1 SD higher log-Lp(a) was 1.07 (95% CI, 0.99-1.15).^39^ In the current analysis restricted to CRIC study participants with prevalent ASCVD followed for a median of 6.6 years, the multivariable-adjusted HR for kidney failure associated with 1 SD higher log-Lp(a) was 1.16 (95% CI, 1.04-1.28). Lp(a) levels increase with lower kidney function.^40^ Although the mechanism behind this association remains unclear, it may involve lower Lp(a) catabolism in the kidney.^40^ Therefore, reverse causation, where individuals with more severe kidney disease have higher Lp(a) levels preceding the development of kidney failure, cannot be excluded. This hypothesis is supported by a prior analysis of the Cohort Study on Chronic Disease of Communities Natural Population in Beijing, Tianjin, and Hebei study.^41^ In this analysis, higher Lp(a) mass concentration was associated with lower eGFR in a cross-sectional analysis. However, higher genetically predicted Lp(a) was associated with higher eGFR in a Mendelian randomization analysis conducted in the same study population, suggesting that Lp(a) may not cause low kidney function. Results from the current study support that Lp(a) may be useful as a risk marker to identify adults with CKD more likely to develop kidney failure.

We conducted multiple subgroup analyses including exploratory outcomes. Therefore, tests of effect modification across subgroups need to be interpreted with caution. Current subgroup analyses suggest that the association of Lp(a) with kidney failure and death may vary by levels of LDL cholesterol and eGFR, respectively. However, we cannot exclude that these findings may be because of chance. These results should be confirmed in future studies.

The current analysis has several strengths. The CRIC study consists of a large, diverse population of adults with mild-to-advanced CKD not on dialysis with a broad range of eGFR levels and has long follow-up for adjudicated ASCVD events, kidney failure, and death. Also, we were able to adjust for several potential confounders, which were assessed using standardized procedures in the CRIC study. We used multiple imputation to include participants with missing data in regression models. The current study also has known and potential limitations. Lp(a) mass concentration, but not molar concentration, is available in the CRIC study. Data on apolipoprotein(a) isoforms were not available. Also, some participant’s characteristics and comorbid conditions were self-reported. The observational design of the current study does not allow determination of the causes of a possible attenuation of the association of Lp(a) with ASCVD risk in participants with versus without diabetes, or whether the association of Lp(a) with kidney failure is causal.

In conclusion, in the current study of adults with CKD who had a history of ASCVD, higher Lp(a) mass concentration was not associated with the risk for recurrent ASCVD events. However, higher Lp(a) mass concentration was associated with an increased risk for kidney failure in an exploratory analysis. These results suggest that Lp(a) mass concentration may not be useful to identify adults with CKD with an increased risk for recurrent ASCVD events, although it may be useful as a risk marker for kidney failure.

## Supplementary Material

Supplementary File (DOCX) Fig S1-S3, Table S1-S5.
