# Supplementary material for: Lipoprotein(a) and the Risk for Recurrent Atherosclerotic Cardiovascular Events Among Adults With CKD: The Chronic Renal Insufficiency Cohort (CRIC) Study
Source: Kidney Med. 2023 May 5;5(7):100648. doi: 10.1016/j.xkme.2023.100648 (PMC10363548; doi:10.1016/j.xkme.2023.100648)
Supplement: Supplementary File (DOCX) — Fig S1-S3, Table S1-S5. [file mmc1.docx]

**SUPPLEMENTAL MATERIAL**

**Lipoprotein(a) and the risk for recurrent atherosclerotic cardiovascular events among adults with chronic kidney disease: The Chronic Renal Insufficiency Cohort Study**

Bharat Poudel^1^, MSPH, Robert S. Rosenson^2^, MD, Shia T. Kent^3^, PhD, Vera Bittner^4^, MD, MSPH, Orlando M. Gutiérrez^1,5^, MD, Amanda H. Anderson^6^, PhD, MPH, Mark Woodward^7,8^, PhD, Elizabeth A. Jackson^4^, MD, MPH, Keri L. Monda^3^, PhD, Archna Bajaj^9^, MD, Lei Huang^1^, PhD, Mayank Kansal^10^, MD, Mahboob Rahman^11^, MD, Jiang He^6^, MD, PhD, Paul Muntner^1^, PhD, Lisandro D. Colantonio^1^, MD, PhD, on behalf of the CRIC Study Investigators*.

^1^ Department of Epidemiology, University of Alabama at Birmingham, Birmingham, AL, USA.

^2^ Mount Sinai Heart, Icahn School of Medicine at Mount Sinai, New York, NY, USA

^3^ Center for Observational Research, Amgen Inc., Thousand Oaks, CA, USA.

^4^ Division of Cardiovascular Disease, Department of Medicine, University of Alabama at Birmingham, Birmingham, AL, USA.

^5^ Division of Nephrology, Department of Medicine, University of Alabama at Birmingham, Birmingham, AL, USA.

^6^ Department of Epidemiology, Tulane University, New Orleans, LA, USA.

^7^ The George Institute for Global Health, Imperial College London, UK.

^8^ The George Institute for Global Health, University of New South Wales, Sydney, Australia.

^9^ Perelman School of Medicine, University of Pennsylvania, Philadelphia, PA, USA.

^10^ Department of Medicine, Division of Cardiology, University of Illinois-Chicago, Chicago, IL, USA.

^11^ Department of Medicine, Case Western Reserve University School of Medicine, University Hospitals of Cleveland, OH, USA.

* Lawrence J. Appel, MD, MPH, Jing Chen, MD, MMSc, MSc, Debbie L Cohen, MD, Harold I. Feldman, MD, MSCE, Alan S. Go, MD, James P. Lash, MD, Robert G. Nelson, MD, PhD, MS, Mahboob Rahman, MD, Panduranga S. Rao, MD, Vallabh O Shah, PhD, MS, Mark L. Unruh, MD, MS.

Table S1. 2021 Chronic Kidney Disease Epidemiology Collaboration (CKD-EPI) equation to calculate eGFR based on creatinine and cystatin C.

|  | 2021 CKD-EPI equation based on creatinine and cystatin C |
| --- | --- |
| For females | eGFR = 135 * min (creatinine / 0.7, 1) ^-0.219^ * max (creatinine / 0.7, 1) ^-0.544^ * min (cystatin C / 0.8, 1) ^-0.323^ * max (cystatin C / 0.8, 1) ^-0.778^ * (0.9961) ^age^ * 0.963 |
| For males | eGFR = 135 * min (creatinine / 0.9, 1) ^-0.144^ * max (creatinine / 0.9, 1) ^-0.544^ * min (cystatin C / 0.8, 1) ^-0.323^ * max (cystatin C / 0.8, 1) ^-0.778^ * (0.9961) ^age^ |

eGFR: estimated glomerular filtration rate.

Reproduced and adapted from: Inker LA, Eneanya ND, Coresh J, Tighiouart H, Wang D, Sang Y, Crews DC, Doria A, Estrella MM, Froissart M, Grams ME, Greene T, Grubb A, Gudnason V, Gutiérrez OM, Kalil R, Karger AB, Mauer M, Navis G, Nelson RG, Poggio ED, Rodby R, Rossing P, Rule AD, Selvin E, Seegmiller JC, Shlipak MG, Torres VE, Yang W, Ballew SH, Couture SJ, Powe NR and Levey AS. New Creatinine- and Cystatin C-Based Equations to Estimate GFR without Race. *N Engl J Med*. 2021 Nov4;385(19);pg1737-1749.^1^

Table S2. Number and percentage of participants with missing data.

| **Study variables** | **N=1,439** |
| --- | --- |
| Age | 0 (0.0%) |
| Sex | 0 (0.0%) |
| Race/ethnicity | 133 (9.2%) |
| Study center | 0 (0.0%) |
| Income | 226 (15.7%) |
| Education less than high school | 0 (0.0%) |
| Current smoking | 0 (0.0%) |
| Physical activity | 4 (0.3%) |
| Body mass index | 6 (0.4%) |
| Diabetes | 0 (0.0%) |
| Systolic blood pressure | 0 (0.0%) |
| Antihypertensive medication use | 8 (0.6%) |
| History of CHD | 0 (0.0%) |
| History of stroke | 0 (0.0%) |
| History of PAD | 0 (0.0%) |
| eGFR | 1 (0.1%) |
| hs-CRP > 2 mg/L | 0 (0.0%) |
| Albumin-to-creatinine ratio | 45 (3.1%) |
| Fibroblast growth factor-23 | 10 (0.7%) |
| HDL cholesterol | 1 (0.1%) |
| Triglycerides | 1 (0.1%) |
| Homocysteine | 137 (9.5%) |
| Aspirin use | 11 (0.8%) |
| Statin use | 11 (0.8%) |
| LDL cholesterol | 3 (0.2%) |
| Non-Lp(a) LDL cholesterol | 3 (0.2%) |
| Cause of death from death certificates | 300 (20.8%) |
| CHD: coronary heart disease; eGFR: estimated glomerular filtration rate; HDL: high-density lipoprotein; hs-CRP: high-sensitivity C-reactive protein; LDL: low-density lipoprotein; MET: metabolic equivalent; PAD: peripheral artery disease. | |

Table S3. Distribution of Lp(a) among participants included in the current analysis.

| **Characteristics** | **Median (25th and 75th percentile) Lp(a), mg/dL** |
| --- | --- |
| Age, years |  |
| <55 | 25.9 (9.0-71.5) |
| 55 to <65 | 31.3 (10.4-73.0) |
| ≥65 | 28.1 (9.5-68.7) |
| Sex |  |
| Females | 36.8 (12.5-77.1) |
| Males | 26.0 (8.6-63.0) |
| Race/ethnicity |  |
| White | 14.2 (6.0-42.2) |
| Black | 51.9 (25.3-86.0) |
| Other | 15.1 (6.5-41.5) |
| Diabetes |  |
| No | 31.0 (10.3-74.6) |
| Yes | 28.5 (9.7-68.7) |
| Systolic blood pressure, mm Hg |  |
| <120 | 22.5 (7.4-62.9) |
| 120 to <140 | 28.1 (10.0-63.6) |
| ≥140 | 38.3 (13.7-84.6) |
| History of CHD |  |
| No | 31.9 (11.1-70.9) |
| Yes | 27.6 (9.3-70.8) |
| History of stroke |  |
| No | 27.8 (9.0-70.6) |
| Yes | 34.1 (12.8-73.9) |
| History of PAD |  |
| No | 25.3 (9.0-63.8) |
| Yes | 33.6 (11.3-75.9) |
| eGFR, ml/min/1.73 m^2^ |  |
| <45 | 33.8 (11.4-76.1) |
| ≥45 | 24.2 (8.0-61.1) |
| ACR, mg/g |  |
| <30 | 27.4 (9.3-67.0) |
| 30 – 300 | 27.0 (8.9-68.2) |
| >300-1000 | 31.0 (10.7-68.7) |
| >1000 | 35.2 (12.3-77.7) |
| LDL cholesterol, mg/dL |  |
| <70 | 16.5 (7.1-39.8) |
| 70 - <100 | 26.4 (9.1-67.1) |
| 100 - <130 | 39.1 (15.3-77.1) |
| ≥130 | 53.1 (16.4-93.9) |
| hs-CRP, mg/L |  |
| <2 | 24.9 (8.4-71.4) |
| ≥2 | 31.9 (10.7-70.8) |
| Aspirin use |  |
| No | 32.1 (10.5-74.4) |
| Yes | 27.9 (9.5-68.1) |
| Statin use |  |
| No | 29.9 (10.9-68.7) |
| Yes | 29.1 (9.5-71.5) |
| ACR: albumin-to-creatinine ratio; CHD: coronary heart disease; eGFR: estimated glomerular filtration rate; hs-CRP: high-sensitivity C-reactive protein; LDL: low-density lipoprotein; Lp(a): lipoprotein(a); PAD: peripheral artery disease. | |

# Table S4. Risk for recurrent atherosclerotic cardiovascular events associated with Lp(a) levels.

|  | **Quartiles of Lp(a)** | | | |  |
| --- | --- | --- | --- | --- | --- |
|  | **Quartile 1** | **Quartile 2** | **Quartile 3** | **Quartile 4** | **p-trend** |
| Lp(a) range, mg/dL | 1.5 to <9.9 | 9.9 to <29.4 | 29.4 to <70.9 | 70.9 to 261.5 |  |
| Events/person-years | 156 / 2,785 | 158 / 2,761 | 169 / 2,592 | 159 / 2,446 |  |
| Rate (95% CI) per 1,000 person-years | 55.9 (47.1-64.7) | 57.1 (48.2-66.0) | 65.2 (55.4-75.1) | 64.9 (54.8-75.0) |  |
| Hazard ratio (95% CI) |  |  |  |  |  |
| Model 1 | 1 (ref) | 0.98 (0.78-1.24) | 1.11 (0.88-1.41) | 1.11 (0.87-1.42) | 0.267 |
| Model 2 | 1 (ref) | 0.99 (0.78-1.25) | 1.06 (0.84-1.36) | 1.00 (0.78-1.29) | 0.778 |
| Model 3 | 1 (ref) | 1.00 (0.79-1.27) | 1.06 (0.83-1.36) | 1.06 (0.82-1.37) | 0.568 |
| CI: confidence interval; Lp(a): lipoprotein(a).  Recurrent atherosclerotic cardiovascular events include myocardial infarction hospitalization, ischemic stroke hospitalization, peripheral artery disease hospitalization, coronary heart disease death or ischemic stroke death.  Model 1 includes adjustment for age, sex, race/ethnicity, Chronic Renal Insufficiency Cohort study center, education, and income.  Model 2 include adjustment for variables in Model 1 and smoking status, physical activity, body mass index, diabetes, systolic blood pressure, antihypertensive medication use, estimated glomerular filtration rate, high-sensitivity C-reactive protein, and albumin-to-creatinine ratio.  Model 3 include adjustment for variables in Model 2 and high-density lipoprotein cholesterol, triglycerides, fibroblast growth factor-23, homocysteine, use of aspirin and statins, and non-Lp(a) low-density lipoprotein cholesterol. | | | | | |

# Table S5. Risk for recurrent cardiovascular events (secondary outcomes), and kidney failure and death (exploratory outcomes) associated with quartiles of Lp(a) levels.

|  | **Quartiles of Lp(a)** | | | |  |
| --- | --- | --- | --- | --- | --- |
|  | **Quartile 1** | **Quartile 2** | **Quartile 3** | **Quartile 4** | **p-trend** |
| Lp(a) range, mg/dL | 1.5 to <9.9 | 9.9 to <29.4 | 29.4 to <70.9 | 70.9 to 261.5 |  |
| **CHD events** |  |  |  |  |  |
| Events/person-years | 131/2,961 | 120/2,988 | 129/2,843 | 111/2,693 |  |
| Rate (95% CI) per 1,000 person-years | 44.2 (36.7-51.8) | 40.0 (32.8-47.2) | 45.4 (37.6-53.2) | 41.3 (33.6-49.0) |  |
| Hazard ratio (95% CI) |  |  |  |  |  |
| Model 1 | 1 (ref) | 0.85 (0.65-1.12) | 1.02 (0.78-1.35) | 0.90 (0.68-1.21) | 0.676 |
| Model 2 | 1 (ref) | 0.83 (0.63-1.10) | 0.96 (0.72-1.27) | 0.81 (0.60-1.09) | 0.273 |
| Model 3 | 1 (ref) | 0.87 (0.66-1.15) | 0.99 (0.74-1.31) | 0.88 (0.65-1.19) | 0.527 |
| **MI hospitalizations** |  |  |  |  |  |
| Events/person-years | 73/2,961 | 66/2,988 | 84/2,843 | 63/2,693 |  |
| Rate (95% CI) per 1,000 person-years | 24.7 (19.0-30.3) | 22.1 (16.8-27.4) | 29.5 (23.2-35.9) | 23.4 (17.6-29.2) |  |
| Hazard ratio (95% CI) |  |  |  |  |  |
| Model 1 | 1 (ref) | 0.86 (0.61-1.21) | 1.29 (0.92-1.82) | 0.98 (0.68-1.41) | 0.773 |
| Model 2 | 1 (ref) | 0.83 (0.59-1.17) | 1.20 (0.85-1.69) | 0.89 (0.62-1.29) | 0.839 |
| Model 3 | 1 (ref) | 0.88 (0.63-1.25) | 1.28 (0.90-1.81) | 1.00 (0.68-1.47) | 0.716 |
| **Ischemic stroke events** |  |  |  |  |  |
| Events/person-years | 23/3,108 | 34/3,095 | 32/3,025 | 38/2,794 |  |
| Rate (95% CI) per 1,000 person-years | 7.3 (4.3-10.3) | 10.7 (7.1-14.4) | 10.5 (6.8-14.1) | 13.7 (9.4-18.0) |  |
| Hazard ratio (95% CI) |  |  |  |  |  |
| Model 1 | 1 (ref) | 1.28 (0.71-2.28) | 1.06 (0.56-2.01) | 1.31 (0.72-2.38) | 0.448 |
| Model 2 | 1 (ref) | 1.30 (0.72-2.35) | 1.11 (0.58-2.12) | 1.25 (0.68-2.29) | 0.594 |
| Model 3 | 1 (ref) | 1.32 (0.73-2.39) | 1.08 (0.56-2.10) | 1.36 (0.73-2.56) | 0.402 |
| **Ischemic stroke hospitalizations** |  |  |  |  |  |
| Events/person-years | 20/3,108 | 30/3,095 | 27/3,025 | 34/2,794 |  |
| Rate (95% CI) per 1,000 person-years | 6.4 (3.6-9.3) | 9.7 (6.2-13.2) | 8.9 (5.6-12.3) | 12.2 (8.1-16.3) |  |
| Hazard ratio (95% CI) |  |  |  |  |  |
| Model 1 | 1 (ref) | 1.33 (0.74-2.37) | 1.01 (0.54-1.89) | 1.29 (0.71-2.36) | 0.597 |
| Model 2 |  | 1.36 (0.75-2.44) | 1.08 (0.57-2.03) | 1.25 (0.68-2.29) | 0.761 |
| Model 3 | 1 (ref) | 1.37 (0.76-2.47) | 1.06 (0.56-2.03) | 1.40 (0.74-2.62) | 0.456 |
| **PAD hospitalizations** |  |  |  |  |  |
| Events/person-years | 38/3,078 | 34/3,069 | 43/2,946 | 38/2,739 |  |
| Rate (95% CI) per 1,000 person-years | 12.3 (8.4-16.3) | 11.1 (7.4-14.8) | 14.6 (10.2-19.0) | 13.9 (9.5-18.3) |  |
| Hazard ratio (95% CI) |  |  |  |  |  |
| Model 1 | 1 (ref) | 0.91 (0.57-1.46) | 1.16 (0.72-1.85) | 1.14 (0.70-1.84) | 0.433 |
| Model 2 | 1 (ref) | 0.94 (0.58-1.52) | 1.15 (0.71-1.85) | 1.02 (0.62-1.68) | 0.816 |
| Model 3 | 1 (ref) | 0.93 (0.57-1.50) | 1.12 (0.69-1.81) | 1.01 (0.61-1.68) | 0.851 |
| **Kidney failure** |  |  |  |  |  |
| Events/person-years | 104/2,960 | 118/2,834 | 149/2,602 | 139/2,443 |  |
| Rate (95% CI) per 1,000 person-years | 35.1 (28.4-41.9) | 41.6 (34.1-49.2) | 57.3 (48.1-66.4) | 56.9 (47.4-66.4) |  |
| Hazard ratio (95% CI) |  |  |  |  |  |
| Model 1 | 1 (ref) | 1.15 (0.88-1.51) | 1.48 (1.13-1.94) | 1.50 (1.14-1.97) | 0.004 |
| Model 2 | 1 (ref) | 1.22 (0.93-1.62) | 1.53 (1.16-2.02) | 1.26 (0.95-1.68) | 0.283 |
| Model 3 | 1 (ref) | 1.24 (0.94-1.65) | 1.61 (1.22-2.14) | 1.33 (1.00-1.77) | 0.161 |
| **Death** |  |  |  |  |  |
| Events/person-years | 199/3,398 | 194/3,394 | 228/3,283 | 224/3,063 |  |
| Rate (95% CI) per 1,000 person-years | 58.6 (50.4-66.7) | 57.2 (49.1-65.2) | 69.4 (60.4-78.5) | 73.1 (63.6-82.7) |  |
| Hazard ratio (95% CI) |  |  |  |  |  |
| Model 1 | 1 (ref) | 0.90 (0.73-1.10) | 1.08 (0.88-1.33) | 1.17 (0.95-1.43) | 0.026 |
| Model 2 | 1 (ref) | 0.88 (0.71-1.07) | 1.02 (0.83-1.26) | 1.02 (0.83-1.26) | 0.397 |
| Model 3 | 1 (ref) | 0.88 (0.72-1.08) | 1.00 (0.81-1.23) | 1.06 (0.85-1.31) | 0.226 |
| CHD: coronary heart disease; CI: confidence interval; Lp(a): lipoprotein(a); MI: myocardial infarction; PAD: peripheral artery disease.  Recurrent cardiovascular events include CHD events (i.e., MI hospitalization or CHD death), MI hospitalizations, ischemic stroke events (i.e., ischemic stroke hospitalization or ischemic stroke death), ischemic stroke hospitalizations, PAD hospitalization. Model 1 includes adjustment for age, sex, race/ethnicity, Chronic Renal Insufficiency Cohort study center, education and income.  Model 2 include adjustment for variables in Model 1 and smoking status, physical activity, body mass index, diabetes, systolic blood pressure, antihypertensive medication use, estimated glomerular filtration rate, high-sensitivity C-reactive protein, and albumin-to-creatinine ratio.  Model 3 include adjustment for variables in Model 2 and high-density lipoprotein cholesterol, triglycerides, fibroblast growth factor-23, homocysteine, use of aspirin and statins, and non-Lp(a) low-density lipoprotein cholesterol. | | | | | |

Figure S1: Flow-chart of CRIC study participants included in the analysis.

All CRIC study participants

N=3,939

2,462 participants excluded who did not have a history of ASCVD.

Participants with a history of ASCVD at baseline

N=1,477

Participants with Lp(a) at baseline

N=1,447

8 participants excluded without follow-up for ASCVD outcome events.

Participants with at least 1 day of follow-up for ascertainment of ASCVD outcome events

N=1,439

ASCVD: atherosclerotic cardiovascular disease; CRIC: Chronic Renal Insufficiency Cohort; Lp(a): lipoprotein(a).

Figure S2. Hazard ratios for kidney failure associated with 1 standard deviation higher log-transformed Lp(a) within sub-groups.


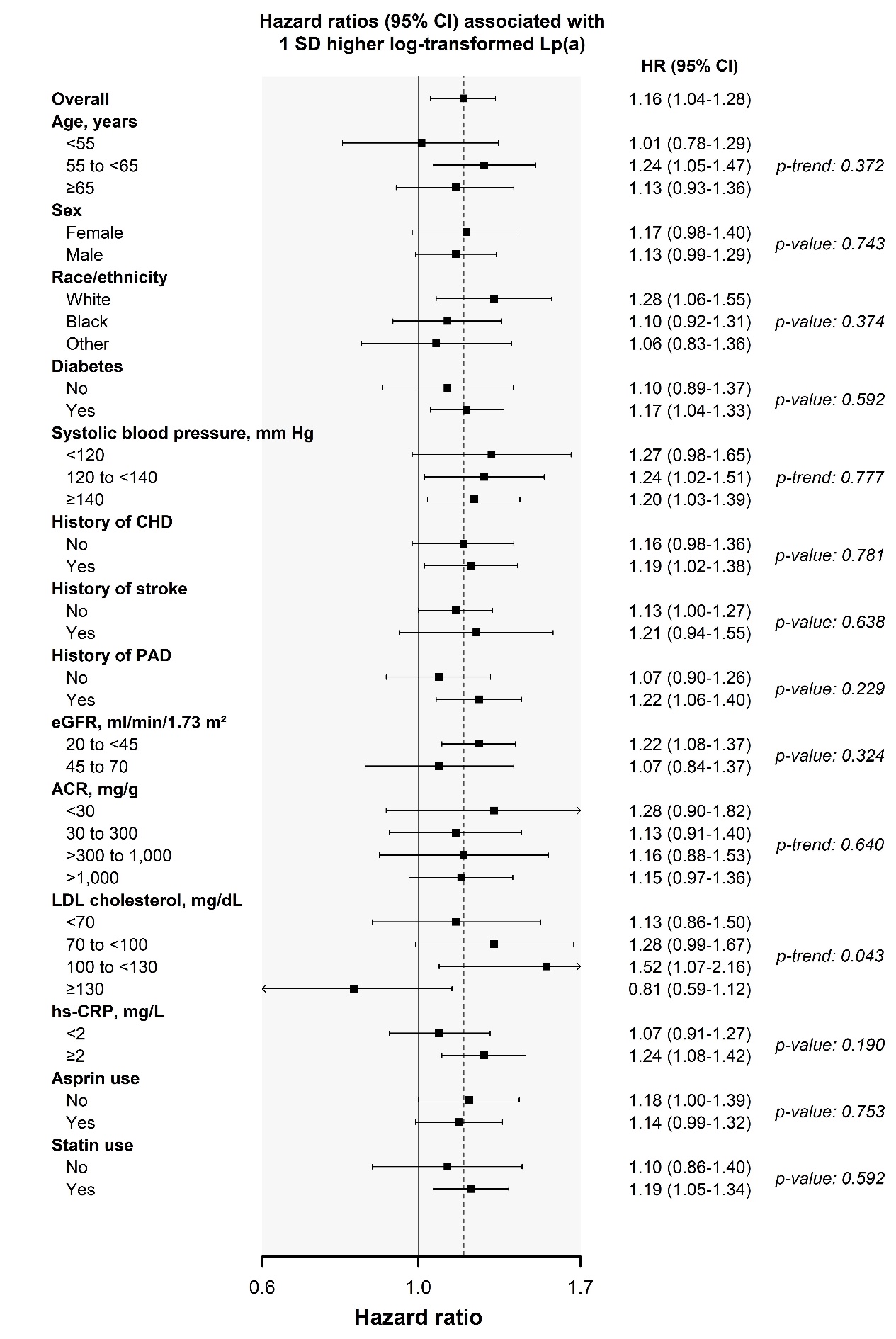


ACR: albumin-to-creatinine-ratio; CHD: coronary heart disease; CI: confidence interval; eGFR: estimated glomerular filtration rate; hs-CRP: high-sensitivity C-reactive protein; HR: hazard ratio; LDL: low-density lipoprotein; Lp(a): lipoprotein(a); PAD: peripheral artery disease; SD: standard deviation.

The SD of log-transformed Lp(a) was equal to 1.3 units, which represents a 3.7 times increase in Lp(a) levels in their original scale.

All hazard ratios were adjusted for age, sex, race/ethnicity, Chronic Renal Insufficiency Cohort study center, education, income, smoking status, physical activity, body mass index, diabetes, systolic blood pressure, antihypertensive medication use, eGFR, high-density lipoprotein cholesterol, triglycerides, high-sensitivity C-reactive protein, albumin-to-creatinine ratio, fibroblast growth factor-23, homocysteine, use of aspirin and statins and non-Lp(a) LDL cholesterol.

Figure S3. Hazard ratios for death associated with 1 standard deviation higher log-transformed Lp(a) within sub-groups.


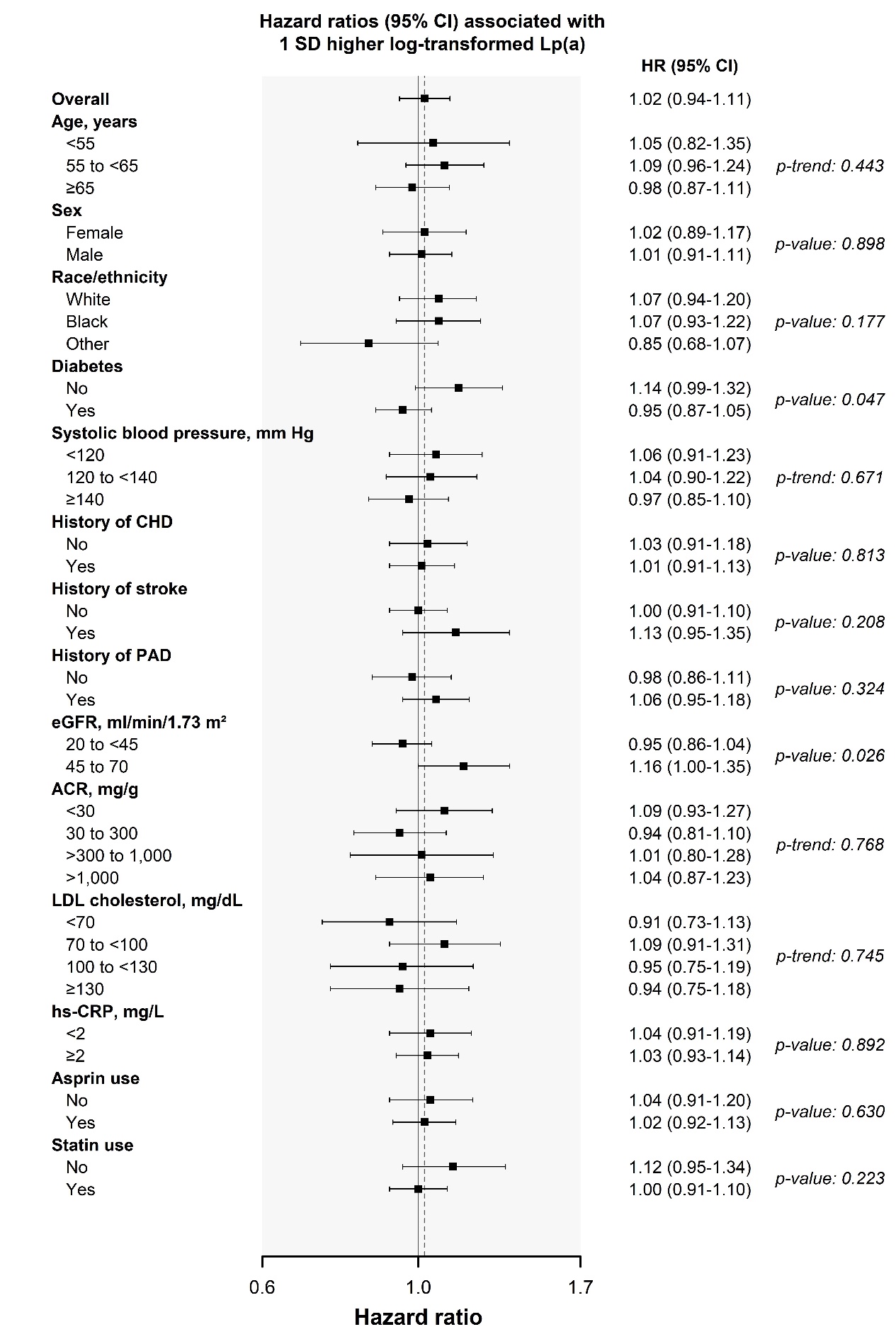


ACR: albumin-to-creatinine-ratio; CHD: coronary heart disease; CI: confidence interval; eGFR: estimated glomerular filtration rate; hs-CRP: high-sensitivity C-reactive protein; HR: hazard ratio; LDL: low-density lipoprotein; Lp(a): lipoprotein(a); PAD: peripheral artery disease; SD: standard deviation.

The SD of log-transformed Lp(a) was equal to 1.3 units, which represents a 3.7 times increase in Lp(a) levels in their original scale.

All hazard ratios were adjusted for age, sex, race/ethnicity, Chronic Renal Insufficiency Cohort study center, education, income, smoking status, physical activity, body mass index, diabetes, systolic blood pressure, antihypertensive medication use, eGFR, high-density lipoprotein cholesterol, triglycerides, high-sensitivity C-reactive protein, albumin-to-creatinine ratio, fibroblast growth factor-23, homocysteine, use of aspirin and statins and non-Lp(a) LDL cholesterol.

**Supplemental References:**

1. Inker LA, Eneanya ND, Coresh J, Tighiouart H, Wang D, Sang Y, Crews DC, Doria A, Estrella MM, Froissart M et al. New Creatinine- and Cystatin C-Based Equations to Estimate GFR without Race. *N Engl J Med*. 2021.
